# Characterisation of the vasodilation effects of DHA and EPA, n-3 PUFAs (fish oils), in rat aorta and mesenteric resistance arteries

**DOI:** 10.1371/journal.pone.0192484

**Published:** 2018-02-02

**Authors:** Roshan Limbu, Graeme S. Cottrell, Alister J. McNeish

**Affiliations:** Reading School of Pharmacy and ICMR, University of Reading, Reading, Berkshire, United Kingdom; "INSERM", FRANCE

## Abstract

**Background and purpose:**

Increasing evidence suggests that the omega-3 polyunsaturated acids (n-3 PUFA), docosahexaenoic acid (DHA) and eicosapentaenoic acid (EPA), are beneficial to cardiovascular health, promoting relaxation of vascular smooth muscle cells and vasodilation. Numerous studies have attempted to study these responses, but to date there has not been a systematic characterisation of both DHA and EPA mediated vasodilation in conduit and resistance arteries. Therefore, we aimed to fully characterise the n-3 PUFA-induced vasodilation pathways in rat aorta and mesenteric artery.

**Methods:**

Wire myography was used to measure the vasomotor responses of freshly dissected rat mesenteric artery and aorta. Arteries were pre-constricted with U46619 and cumulative concentrations of either DHA or EPA (10 nM-30 μM) were added. The mechanisms by which n-3 PUFA relaxed arteries were investigated using inhibitors of vasodilator pathways, which include: nitric oxide synthase (NOS; L-NAME), cycloxygenase (COX; indomethacin), cytochrome P450 epoxygenase (CYP450; clotrimazole); and calcium-activated potassium channels (K_Ca_), SK_Ca_ (apamin), IK_Ca_ (TRAM-34) and BK_Ca_ (paxilline).

**Results:**

Both DHA- and EPA-induced relaxations were partially inhibited following endothelium removal in rat mesenteric arteries. Similarly, in aorta EPA-induced relaxation was partially suppressed due to endothelium removal. CYP450 also contributed to EPA-induced relaxation in mesenteric artery. Inhibition of IK_Ca_ partially attenuated DHA-induced relaxation in aorta and mesenteric artery along with EPA-induced relaxation in mesenteric artery. Furthermore, this inhibition of DHA- and EPA-induced relaxation was increased following the additional blockade of BK_Ca_ in these arteries.

**Conclusions:**

This study provides evidence of heterogeneity in the vasodilation mechanisms of DHA and EPA in different vascular beds. Our data also demonstrates that endothelium removal has little effect on relaxations produced by either PUFA. We demonstrate IK_Ca_ and BK_Ca_ are involved in DHA-induced relaxation in rat aorta and mesenteric artery; and EPA-induced relaxation in rat mesenteric artery only. CYP450 derived metabolites of EPA may also be involved in BK_Ca_ dependent relaxation. To our knowledge this is the first study indicating the involvement of IK_Ca_ in n-3 PUFA mediated relaxation.

## Introduction

Cardiovascular diseases (CVDs) are the leading cause of deaths worldwide and according to the World Health Organisation, CVDs account for up to 31% of all deaths globally. One of the major risk factors associated with CVDs is endothelial and vascular dysfunction which causes impairment of vascular relaxation and reactivity [1]. Endothelium lines the interior surface of blood vessels and has a critical role in the production of various vasodilators such as nitric oxide (NO), prostaglandins, endothelium-dependent hyperpolarization (EDH) and endothelium-derived hyperpolarization factors (EDHFs) that include; hydrogen peroxide and cytochrome P450 (CYP450) metabolites of arachidonic acid (AA) [2–6].

The cardioprotective effects of omega-3 long chain polyunsaturated fatty acids (n-3 PUFAs) or “fish oils” were first identified in Greenland and Japanese populations where the mortality rate from CVDs were significantly less compared to Western populations [7, 8]. These beneficial effects were attributed to high consumption of fish; subsequently clinical and epidemiological studies on n-3 PUFAs reported therapeutic benefits to health [9]. The beneficial effects of n-3 PUFAs include providing protective cardiovascular effects, enhancing brain function, attenuating the risk of cancer, and inhibiting inflammation [10–12]. There are three main types of n-3 PUFAs found in fish: alpha linolenic acid (ALA, 18:3), eicosapentaenoic acid (EPA, 20:5), and docosahexaenoic acid (DHA, 22:6) [13]. DHA and EPA are primarily associated with the beneficial effects of n-3 PUFAs, including vasodilation [14].

Vascular studies have reported that dietary fats can affect endothelial function and overall vascular tone [15]. For example, AA is an omega-6 PUFA involved in numerous signalling pathways including vasodilation—reviewed in [4, 6, 16–18]. Different enzymes are involved in the production of metabolites of AA, also known as eicosanoids, these include; cycloxygenase (COX)-derived series-2 prostaglandins (e.g. PGI_2_) and cytochrome P450 epoxygenase (CYP450)-derived epoxyeicosatrienoic acids (EETs) both of which are known to evoke vasodilation [16, 17]. Similar to AA, n-3 PUFAs can also be found as free fatty acids and can be released from membrane phospholipids via the activity of phospholipase A2 (PLA2) [19, 20]. n-3 PUFAs compete with AA as substrates for many enzymes including those involved in the production of AA-derived eicosanoids [21, 22]. For example, EPA and DHA produce COX metabolites (series-3 PGs), CYP450 metabolites known as epoxyeicosatetraenoic acids (EpETEs) derived from EPA [23] and epoxydocosapentaenoic acids (EDPs) derived from DHA [22] which are all involved in vasodilation [24–26].

n-3 PUFAs can improve endothelial function and vascular reactivity in both healthy volunteers and patients suffering from cardiovascular disorders [27–29]. These studies indicated an increased arterial vasodilatation following the dietary inclusion of n-3 PUFAs; the mechanisms involved can differ depending upon the n-3 PUFA studied [28]. One mechanism proposed to be involved in these responses is the improved bioavailability of NO [29]. However, n-3 PUFAs also compete with AA for various enzymes involved in vasodilation [30], indicating that these vasodilator pathways also contribute to n-3 PUFA mediated relaxation. For example, EDPs derived from DHA metabolism by CYP450s are involved in vasodilation of porcine coronary arteries [25]. Similar to AA-derived EETs, these EDPs were reported to activate large conductance calcium activated potassium channels (BK_Ca_) resulting in hyperpolarization and relaxation of vascular smooth muscle cells (VSMCs). COX metabolites of EPA are also reported to be involved in n-3 PUFA mediated vasodilation [24]. These studies indicate that n-3 PUFAs evoke relaxation through an endothelium-dependent mechanism, but there is also evidence that they may act directly on VSMCs via uncharacterized mechanisms [27].

Few studies have looked in depth into the individual vasodilation mechanisms of DHA and EPA and different mechanisms are reported to be involved, depending upon the type of artery and n-3 PUFA studied. Therefore, this study focused on the detailed characterisation of common vasodilation pathways including NO, COX, CYP450 and EDH-like responses in the individual vasodilator effects of DHA and EPA. We conducted these studies in a conduit artery (aorta) and a resistance artery (mesenteric artery) of rats as the vasodilator mechanisms in these artery types show considerable heterogeneity; the NO pathway dominating in conduit arteries and a greater contribution of EDH in resistance arteries [6, 31]. We confirm the role of BK_Ca_ and provide evidence of a novel role for intermediate K_Ca_ (IK_Ca_) channels in relaxation mediated by DHA in rat aorta and mesenteric artery along with EPA-induced relaxation in rat mesenteric artery.

## Methods

Male Wistar Kyoto (WKY, 8–12 weeks, 200–300 g) rats were killed according to schedule one of the Animals (Scientific Procedures) Act 1986 and thus was given an ethical approval waiver by the University of Reading Animal Welfare and Ethical Review Board (AWERB). To ensure death, an inhaled overdose of isoflurane was immediately followed by cervical dislocation. The aortic and mesenteric vascular beds were dissected from WKY rats and immediately placed in ice-cold isotonic Krebs solution containing (mM): CaCl_2_, 2.5; glucose, 11; KCl, 3.6; KH_2_PO_4_, 1.2; MgSO_4_.7H_2_O, 1.2; NaCl, 118 and NaHCO_3_, 24. Segments of aorta and third order mesenteric arteries (~2 mm of length) were mounted in Mulvany-Halpern wire myograph (Danish MyoTechnology, 620M). The tissues were immersed in Krebs solution bubbled with 95% O_2_/5% CO_2_ and subjected to zero tension followed by equilibration at 37°C for 20 minutes. The tissues were then stretched to a standardized tension of 7–13 mN (aorta) and ~3 mN (mesenteric artery) according to the DMT normalization module in Labchart 7. Tension was measured using isometric force transducer connected to PowerLab (ML846; AD Instruments, UK) and a computer running Labchart 7 software (AD Instruments, UK). All arteries were tested for functional endothelium by preconstricting them with the thromboxane A_2_ receptor (TP) agonist, U46619 (5–100 nM) followed by the addition of acetylcholine (ACh, 1 μM) to induce vascular relaxation. Arteries that exhibited >90% relaxation were considered to have viable endothelium. A stable sub-maximal tone (~ 50–80% of maximum) was elicited with U46619 (5–100 nM) and n-3 PUFA mediated relaxations were investigated through cumulative addition with increasing concentrations (10 nM—30 μM). In some experiments the endothelium was removed to assess its role in n-3 PUFA mediated relaxation by gently rubbing the inner layer of the arteries with either a stainless steel wire (Diameter: 250 μm) (aorta) or gold plated tungsten wire (Diameter: 25 μm) (mesenteric artery). Arteries with <10% relaxation to ACh (1 μM) were considered to have functional removal of endothelium. Following the control concentration response curve, in the same arterial rings, inhibitors were incubated for at least 20 mins before preconstricting to a similar level of tone as was achieved in the control experiment by adjusting the concentration of U46619 (5–100 nM), if necessary. The role of eNOS, COX, CYP450 and EDH-like responses were assessed on DHA- and EPA-mediated relaxation. NO, COX and CYP450 pathways were blocked by: the selective nitric oxide synthase inhibitor L-NAME (300 μM), the selective cycloxygenase inhibitor indomethacin (10 μM) and the non-selective CYP450 inhibitor clotrimazole (1 μM) respectively. In order to investigate the EDH response, experiments were conducted in the presence of L-NAME to prevent the effect of basal NO release or NO mediated responses. The K_Ca_ channels involved in EDH responses were inhibited with the specific blockers, apamin (SK_Ca_ blocker, 50 nM), TRAM-34 (IK_Ca_ blocker, 1 μM) and paxilline (BK_Ca_ blocker, 1 μM). Often EDH-like responses require blockade of all 3 subtypes of K_Ca_ generally the combination of SK_Ca_ and IK_Ca_ is sufficient to block this pathway [6]. We initially added apamin which failed to affect responses; therefore, we then assessed the combination apamin and TRAM-34 which would elucidate the role of IK_Ca_. As residual relaxation was observed following this combination, the further contribution of BK_Ca_ (and thus the total EDH/EDHF component of relaxation) was assessed by adding paxilline to this blocking cocktail.

### Data analysis and statistical procedures

Results are expressed as mean±SEM of *n* experiments, where *n* refers to the number of biological replicates each obtained from a separate animal. Data analysis was carried out using GraphPad Prism 5 (v5.0, GraphPad Software, San Diego, CA, USA). Relaxation response was measured as percentage reduction of the stable tone induced by U46619 (5–100 nM). One-way analysis of variance (ANOVA) and Bonferroni’s post-hoc test or two-tailed Student’s t-test (as appropriate) were used for statistical comparison of the concentration response curves in GraphPad Prism 5 (GraphPad, USA). P-value of < 0.05 was considered as being statistically significant.

### Drugs, chemicals, reagents and other materials

All inhibitors were obtained from Sigma (Poole, UK). Salts for Krebs solution were obtained from Fisher Scientific (Loughborough, UK) with the exception of CaCl_2_ and MgSO_4_.7H_2_O which were acquired from Sigma. Apamin, L-NAME (L-N^G^-Nitroarginine methyl ester) and acetylcholine were dissolved in distilled water. Clotrimazole (1-[(2-Chlorophenyl)(diphenyl)methyl]-1H-imidazole), indomethacin (2-{1-[(4-Chlorophenyl)carbonyl]-5-methoxy-2-methyl-1H-indol-3-yl}acetic acid), paxilline ((2R, 4bS, 6aS, 12bS, 12cR, 14aS)-4b-hydroxy-2-(1-hydroxy-1-methylethyl)-12b, 12c-dimethyl-5, 6, 6a, 7, 12, 12b, 12c, 13, 14, 14a-decahydro-2H-chromeno [5',6': 6,7] indeno [1,2-b] indol-3(4bH)-one), TRAM-34 (1-[(2-chlorophenyl)diphenylmethyl]-1*H*-pyrazole) and U46619 (9,11-dideoxy-9α,11α-methanoepoxy PGF2α) were dissolved in 100% dimethyl sulphoxide (DMSO). DHA and EPA were dissolved in 100% ethanol and subsequent dilutions were carried out in distilled deionized water. All stock drugs were prepared at 10 mM with the exception of L-NAME (100 mM), indomethacin (100 mM) and apamin (100 μM).

## Results

For all experimental groups EC_50_ and maximum relaxation (E_max_ %) to each n-3 PUFA were calculated and can be found in supplemental materials (Table A in S1 File). A pooled analysis of control curves for DHA and EPA mediated relaxation (Table B in S1 File) demonstrated that there was no difference in the E_max_ but that DHA was significantly more potent at evoking relaxation in both mesenteric artery (*n* = 19–20, P<0.05) and aorta (*n* = 17–18, P<0.05; Table B in S1 File).

### Role of endothelium in n-3 PUFA-dependent relaxation of rat mesenteric artery and aorta

**Fig 1:**
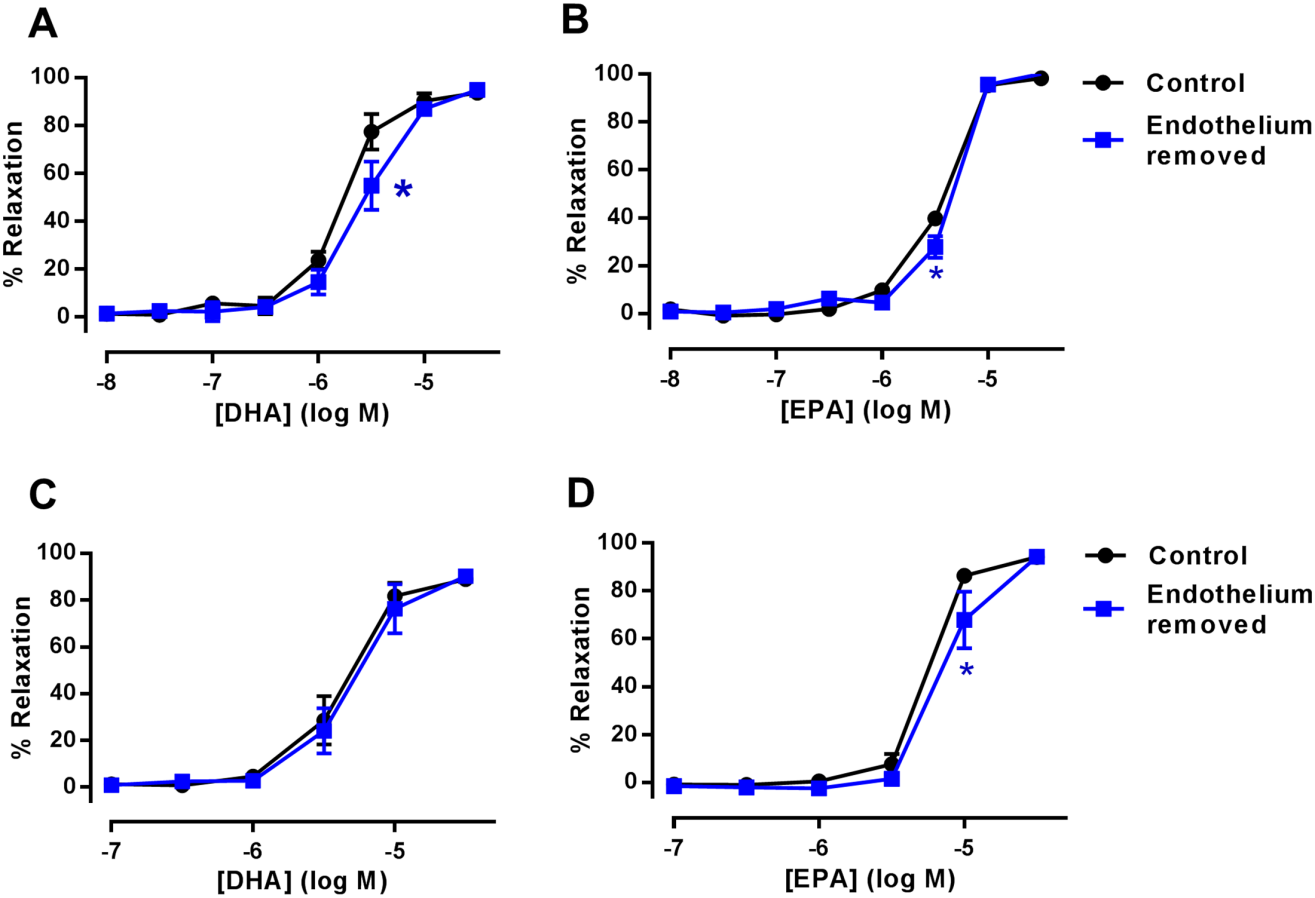
The effect of endothelium removal in n-3 PUFA-induced relaxation of rat arteries preconstricted with U46619. (A) DHA (*n* = 5) and (B) EPA-induced relaxation of rat mesenteric artery following endothelium removal (*n* = 6). (C) DHA (*n* = 7) and (D) EPA-induced relaxation of rat aorta following endothelium removal (*n* = 6). Data are expressed as mean±SEM. * Indicates P<0.05, significant difference from control curve was assessed by one-way ANOVA followed by Bonferroni post-test.

Endothelium has a critical role in maintaining vascular homeostasis therefore we assessed the contribution of endothelium to DHA and EPA mediated vasodilation. Both n-3 PUFAs evoked concentration dependent relaxation of mesenteric artery and aorta. Relaxations to both DHA and EPA were partially inhibited following endothelium removal in rat mesenteric arteries (Fig 1A and 1B) (*n* = 5–6, P<0.05). Similarly, in aorta EPA-induced relaxation was partially suppressed following endothelium removal (Fig 1D, *n* = 6, P<0.05). However, DHA-induced relaxation was unaffected by the removal of endothelium in aorta (Fig 1C, *n* = 7).

### Role of eNOS and COX in n-3 PUFA-dependent relaxation of rat mesenteric artery and aorta

**Fig 2:**
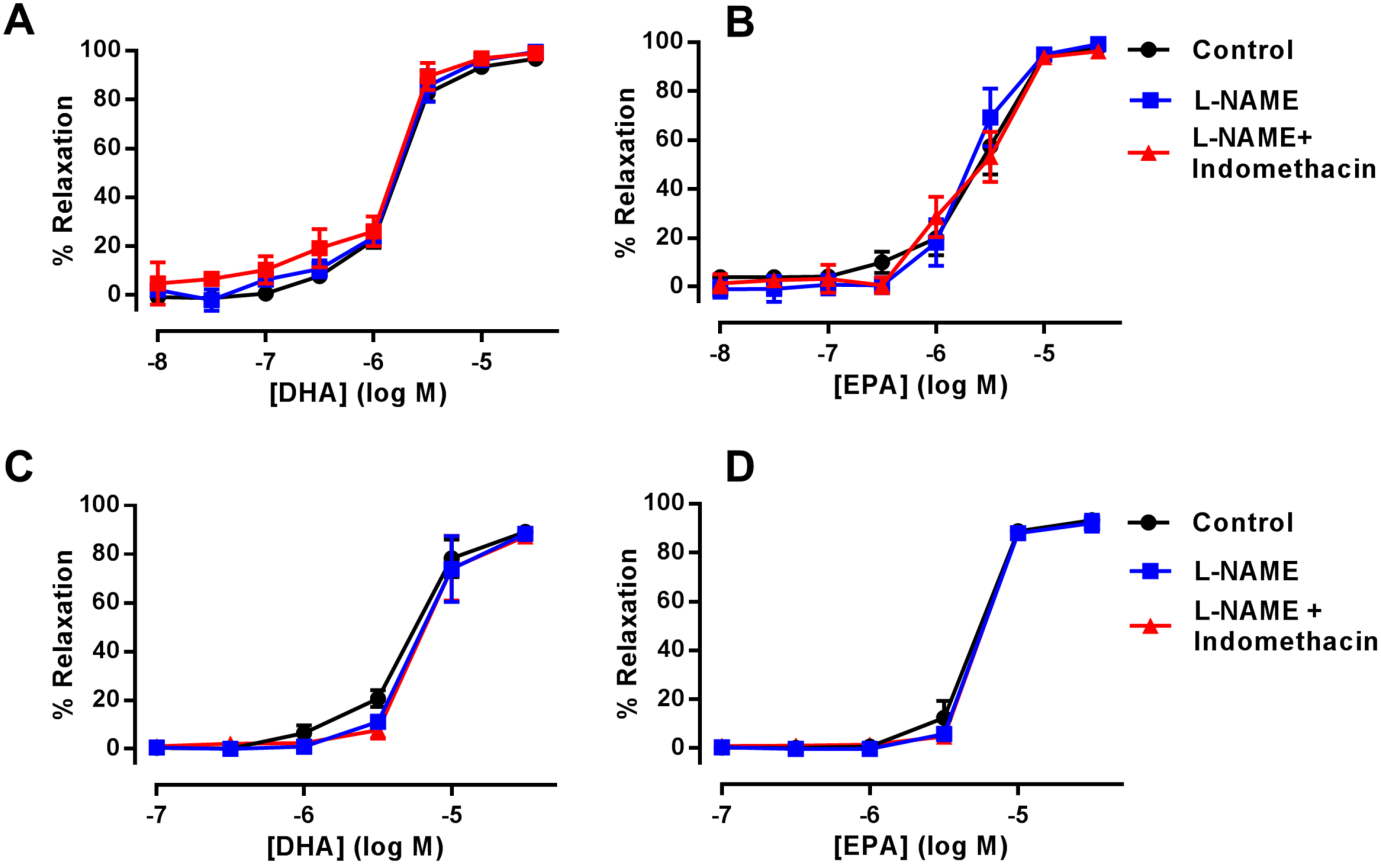
The effects of L-NAME and indomethacin in n-3 PUFA-induced relaxation of rat arteries preconstricted with U46619. (A) DHA- and (B) EPA-induced relaxation of rat mesenteric artery in the presence of L-NAME (300 μM) and subsequent addition of indomethacin (10 μM) (*n* = 5). (C) DHA- and (D) EPA-induced relaxation of rat aorta in the presence of L-NAME and subsequent addition of indomethacin (*n* = 5). Data are expressed as mean±SEM. * Indicates P<0.05, significant difference from control curve was assessed by one-way ANOVA followed by Bonferroni post-test.

In rat mesenteric artery, DHA and EPA-induced relaxation are unaffected by the inhibition of eNOS (L-NAME, 300 μM) and the additional inhibition of COX (indomethacin, 10 μM) (Fig 2A and 2B) (*n* = 5). Similarly, in rat aorta both DHA- and EPA-induced relaxations were not altered in the presence of L-NAME alone, or in combination with indomethacin (Fig 2C and 2D) (*n* = 5).

### Role of CYP450 in n-3 PUFA-dependent relaxation of rat mesenteric artery and aorta

**Fig 3:**
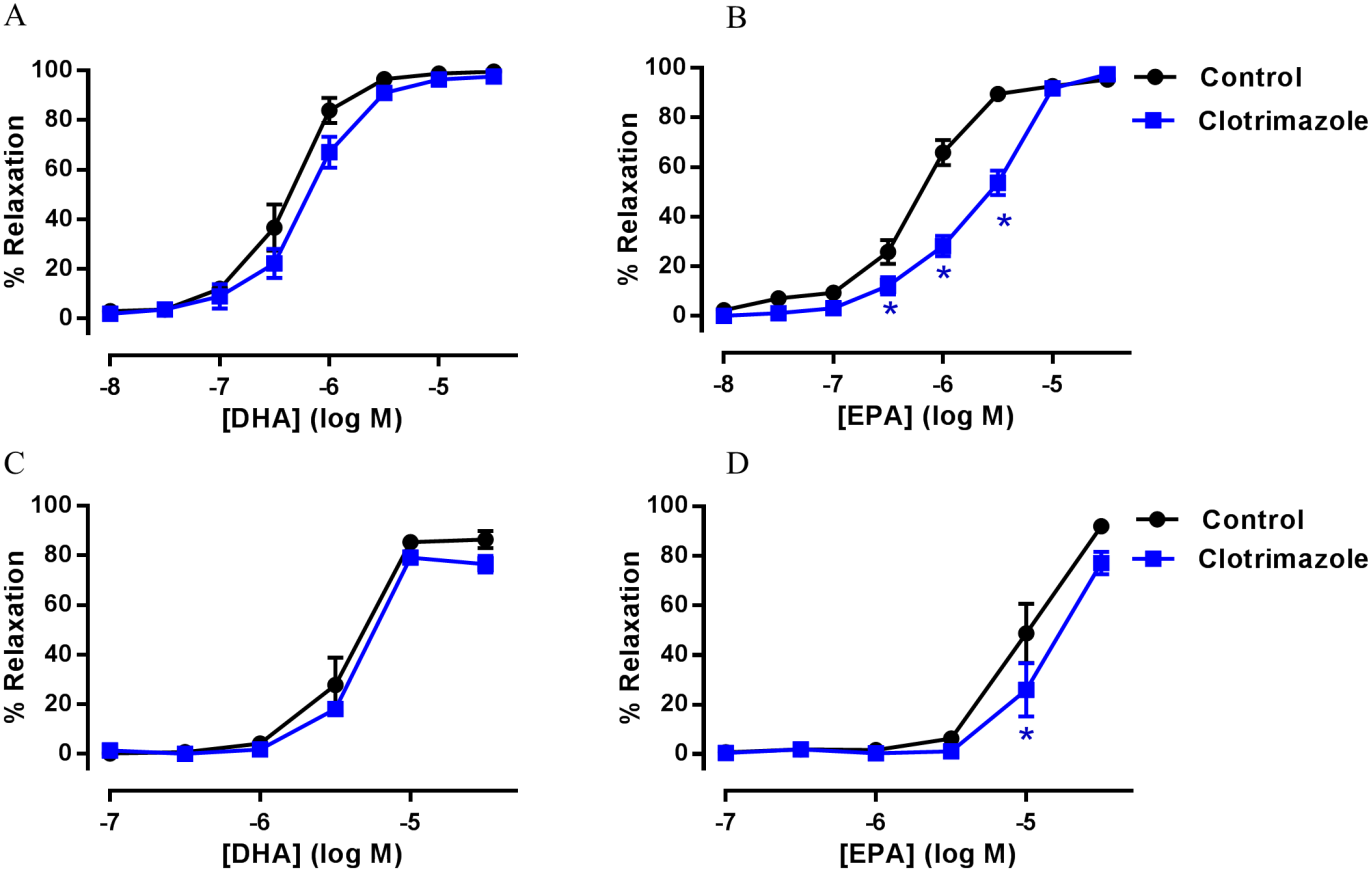
The effect of clotrimazole in n-3 PUFA-induced relaxation of rat arteries preconstricted with U46619. (A) DHA- and (B) EPA-induced relaxation of rat mesenteric artery in the presence of clotrimazole (1 μM) (*n* = 5–6). (C) DHA- and (D) EPA-induced relaxation of rat aorta in the presence of clotrimazole (*n* = 5). Data are expressed as mean±SEM. * Indicates P<0.05, significant difference from control curve was assessed by one-way ANOVA followed by Bonferroni post-test.

The effect of CYP450 epoxygenase inhibition on n-3 PUFA-mediated relaxation was investigated in rat mesenteric artery and aorta. Fig 3A and 3C demonstrate that non-selective inhibition of CYP450 (clotrimazole, 1 μM) did not modify the relaxant effects of DHA in rat mesenteric artery and aorta (*n* = 5 and 6, respectively). In contrast, EPA-induced relaxation in rat mesenteric artery and aorta were both partially inhibited with clotrimazole (1 μM) as shown in Fig 3B and 3D (*n* = 5, P<0.05). These findings indicate that CYP450 epoxygenase metabolites of EPA are involved in vasodilation.

### Role of K_Ca_ channels in n-3 PUFA-dependent relaxation of rat mesenteric artery

**Fig 4:**
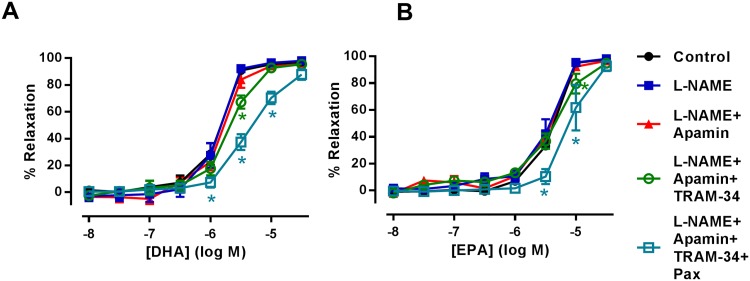
The effects of inhibiting K_Ca_ channels in n-3 PUFA-induced relaxation of rat mesenteric artery preconstricted with U46619. (A) DHA- and (B) EPA-induced relaxation of rat mesenteric artery in the presence of L-NAME (300 μM) followed by the subsequent addition of K_Ca_ inhibitors; apamin (50 μM), TRAM-34 (1 μM) and paxilline (Pax, 1 μM) (*n* = 5). Data are expressed as mean±SEM. *Indicates P<0.05, significant difference from control curve assessed by one-way ANOVA followed by Bonferroni post-test.

The EDH pathway is an integral component of endothelium-dependent relaxation in resistance arteries [32]. This pathway was investigated through the blockade of K_Ca_ channels responsible for subsequent hyperpolarization and relaxation of VSMCs. Pre-treatment of rat mesenteric artery with the combination of L-NAME and the SK_Ca_ channel inhibitor, apamin (50 nM), did not modify DHA-induced relaxation. However, additional inhibition of IK_Ca_ with TRAM-34 (1 μM) partially inhibited this relaxation (*n* = 5, P<0.05) (Fig 4A). Subsequent addition of the BK_Ca_ inhibitor, paxilline (1 μM) further inhibited DHA-induced relaxation (Fig 4A) (P<0.05). Consistent with these findings using identical experimental conditions, inhibition of IK_Ca_ and BK_Ca_ channels also inhibited EPA-induced relaxations in rat mesenteric artery (Fig 4B *n* = 5, P<0.05).

### Role of K_Ca_ channels in n-3 PUFA-dependent relaxation of rat aorta

**Fig 5:**
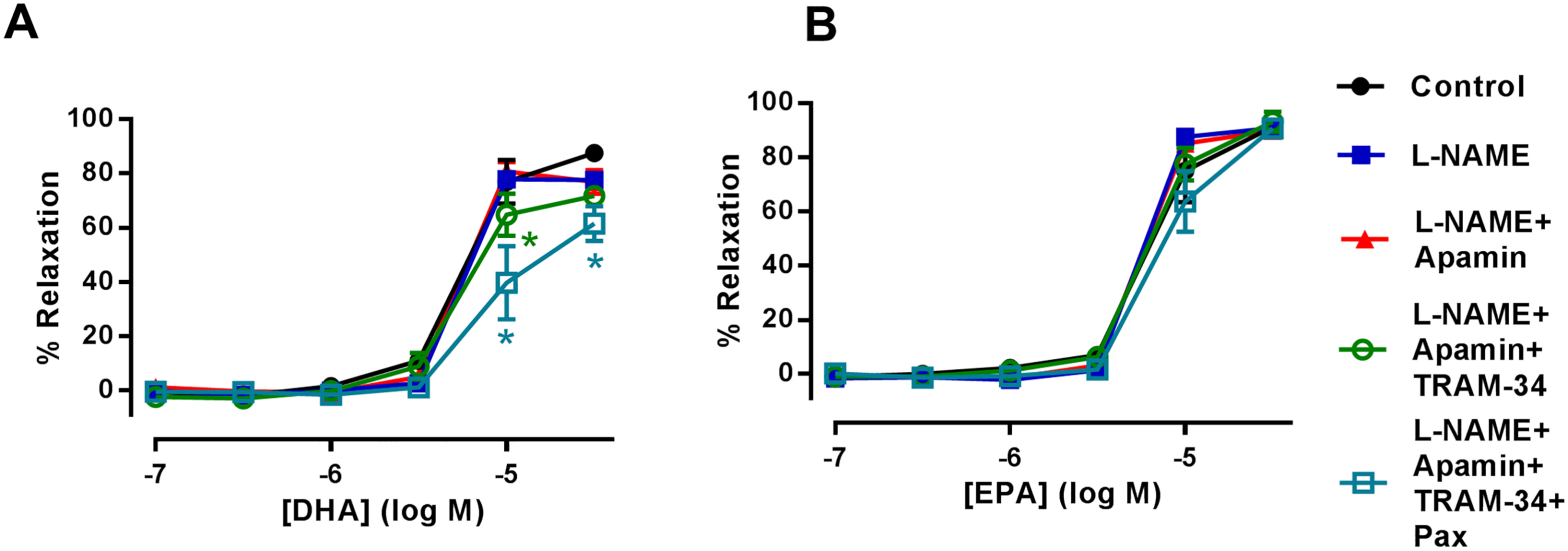
The effects of inhibiting K_Ca_ channels in n-3 PUFA-induced relaxation of rat aorta preconstricted with U46619. (A) DHA- and (B) EPA-induced relaxation of rat aorta in the presence of L-NAME (300 μM) followed by the subsequent addition of K_Ca_ inhibitors; apamin (50 μM), TRAM-34 (1 μM) and paxilline (Pax, 1 μM) (*n* = 5). Data are expressed as mean ± SEM. * Indicates P<0.05, significant difference from control curve was assessed by one-way ANOVA followed by Bonferroni post-test.

While EDH is not the predominant vasodilation mechanism in the aorta, K_Ca_ channels are present and can affect dilator responses [33–36]. Combined inhibition of eNOS with L-NAME (300 μM) and SK_Ca_ with apamin (50 nM) did not modify DHA-induced relaxation (Fig 5A). However, subsequent inhibition of IK_Ca_ with TRAM-34 (1 μM) led to partial attenuation of DHA-induced relaxation which was enhanced upon additional inhibition of BK_Ca_ with paxilline (1 μM) (*n* = 5, P<0.05). In contrast, combined inhibition of eNOS, SK_Ca_, IK_Ca_ and BK_Ca_ did not affect the EPA-induced relaxation of rat aorta (Fig 5B) (*n* = 5).

## Discussion

CVDs are associated with the impairment of vasodilation mechanisms in arteries [14, 37]. The n-3 PUFAs, EPA and DHA, found in fish and supplements are reported to improve vasodilation through different mechanisms that promote endothelial function and vascular reactivity [27]. Our study characterised n-3 PUFA-induced relaxation at concentrations of free fatty acid (100 nM-30 μM) that are achievable in human plasma following a n-PUFA rich meal (~70 μM) [38]. We did this in both resistance and conduit arteries of rats since studies suggest that the vasodilation mechanisms can differ depending upon the type of artery [31]. Conduit arteries are the larger elastic blood vessels that are mainly involved in the distribution of blood [39] whereas arteries with the lumen diameter of <300 μm are classed as resistance arteries and are critical in the regulation of blood pressure [40, 41]; vasodilation is predominantly mediated by NO in conduit arteries while in resistance arteries EDH mechanisms also contribute to relaxation [31, 36]. Therefore, we investigated both types of blood vessel to fully understand the mechanisms involved with n-3 PUFA mediated relaxation.

Endothelium has an important role in the regulation of vascular tone since it is involved in the production of various vasodilators including NO, PGI_2_ and EETs, along with the transmission of endothelial hyperpolarization to VSMCs via myoendothelial gap junctions [2–5]. We investigated the effect of endothelial removal in n-3 PUFA mediated relaxation of rat aorta and mesenteric arteries. Our findings indicate that endothelial removal causes partial attenuation in both DHA- and EPA-induced relaxation of rat mesenteric artery (Fig 1A and 1B). Similarly, this inhibitory effect was also observed with EPA-induced relaxation of rat aorta (Fig 1D). This is consistent with numerous reports suggesting that n-3 PUFAs can improve endothelial function and augment endothelium dependent relaxation [15, 27–29, 42]. However, relaxation was only partially inhibited following removal of endothelium and a large residual relaxation remained indicating that the vasodilator effect of n-3 PUFAs is primarily endothelium-independent. Furthermore, as DHA-induced relaxation remained unaltered following endothelium removal in the aorta (Fig 1C), there is heterogeneity in the vasodilator mechanisms of DHA between conduit and resistance arteries.

A large body of evidence exists demonstrating that n-3 PUFAs can evoke endothelium-dependent NO-mediated relaxation. For example, EPA was reported to induce calcium-independent increases in NO resulting in relaxation of bovine coronary arteries [43]. Similarly, DHA was also reported to enhance IL-1β-mediated NO production in VSMCs derived from rat aorta [44]. Elevation of eNOS mRNA and protein levels in isolated aortic tissues have been suggested as a mechanism of n-3 PUFA-induced NO production [45]. Clinical studies have validated these findings, indicating that long-term EPA treatment in patients with coronary artery disease improved both NO-dependent and -independent vasodilation [29]. However, our results demonstrate that inhibition of eNOS did not affect DHA- and EPA-induced relaxation in rat mesenteric artery and aorta (Fig 2A–2D). Other groups have reported a lack of effect of n-3 PUFAs on NO mediated relaxation. For example a study investigating the effect of DHA supplementation in orchidectomized animals [46] found that vasodilator responses and NO levels were significantly lower in orchidectomized rats compared to the control rats and that DHA normalized these levels [46]. However DHA-supplemented control rats did not have altered levels of NO in aortic segments. Additionally, in placental tissue derived from pregnancy-induced hypertensive rats with impaired vasodilator responses, n-3 PUFA supplementation led to an increase in eNOS levels [47]. Therefore, it is possible that the beneficial effects of n-3 PUFAs on NO production can only be observed in conditions where NO bioavailability is compromised. a recent meta-analysis indicated that the hypotensive effects of n-3 PUFAs are only observed in hypertensive individuals but not in healthy volunteers [48]. We believe our study indicates that n-3 PUFAs do not affect eNOS-mediated relaxations when acutely applied to arterial tissue from healthy animals and highlights that care must be taken when comparing n-3 PUFA effects between species and disease models.

Both DHA and EPA compete with AA as substrates for COX enzymes resulting in the production of vasoactive metabolites and clinical evidence demonstrates there is altered prostanoid production as a result of fish oil supplementation in humans [49]. Furthermore, n-3 PUFAs inhibit noradrenaline- and angiotensin II-induced vasoconstriction of human forearm resistance vessels, an effect that is sensitive to COX blockade [49]. However, we found no evidence of COX-derived metabolites contributing to n-3 PUFA-mediated relaxation in either mesenteric artery or aorta (Fig 2). This is in contrast to previous studies by Engler *et al* where COX-derived metabolites of DHA and EPA were reported to be involved in relaxation of rat aorta [24, 50]. In these studies, relaxations were sensitive to the blockade of COX and adenosine triphosphate-sensitive potassium channels (K_ATP_); consistent with studies indicating that AA-derived prostanoids activate vascular K_ATP_ [51]. This discrepancy might have occurred due to significant methodological differences between our laboratory and that of Engler *et al* [24, 50], which include the use of a different vasoconstrictor (noradrenaline) and the use of considerably larger and older WKY rats by the Engler group [16–17 weeks, 355±11 g). There is evidence indicating that ageing is associated with endothelial dysfunction and therefore is a risk factor for CVDs [52]. Various studies have indicated that ageing can evoke biochemical changes in the blood vessels resulting in impairment of NO production and PGI_2_-induced relaxation [53–57]. Therefore, it is possible that ageing could also affect the mechanisms involved with n-3 PUFA-induced vasodilation [58]; for example, the COX metabolites of n-3 PUFAs may have a more profound effect in improving the impaired endothelial function in older rats and this may partly explain the discrepancy. However, our findings are consistent with another study where combined inhibition of COX and eNOS did not modify DHA-induced relaxation of U46619-constricted rat aorta [59]. This study was conducted with rats of the same age and weight as used in our study. Further investigation is required to examine if different vasoconstrictor agonists and age groups of WKY rats alter the mechanisms underlying n-3 PUFA mediated vasodilation.

In addition to the reported effects on NOS and COX mediated relaxations, n-3 PUFA compete with AA as substrates for CYP450, resulting in the production of different vasoactive metabolites in arteries [22, 25, 60]. In porcine coronary arteries, DHA-derived CYP450 metabolites, EDPs, were reported to activate BK_Ca_ channels found in VSMCs resulting in hyperpolarization and vasodilation [25]. Furthermore, CYP450 metabolites of EPA, 17(18)- EpETEs, relax pulmonary artery through activation of BK_Ca_ [60]. Therefore, we investigated the role of CYP450 epoxygenase in rat mesenteric artery and aorta with n-3 PUFAs. Our findings demonstrate that inhibition of CYP450 epoxygenase did not affect DHA-induced relaxation in either artery (Fig 3A and 3C). In contrast, EPA-induced relaxation was partially attenuated by inhibition of CYP450 in both arteries (Fig 3B and 3D). These findings again indicate heterogeneity in the vasodilation mechanisms of n-3 PUFAs. The lack of effect on DHA mediated relaxation and the large proportion of relaxation remaining following the blockade of CYP450 in EPA mediated relaxation, suggests other mechanisms are also involved. Therefore, our findings suggest that that n-3 PUFAs do not necessarily require CYP450 metabolism to induce full vascular relaxation. However, we cannot eliminate the possibility that metabolism by other enzymes, such as lipoxygenase, could contribute to these relaxation responses [61].

In this study, we also investigated if mechanisms of EDH-mediated relaxation could be involved with the vasodilation effect of n-3 PUFAs. EDH is an important vasodilation pathway, especially in smaller resistance arteries, that involves endothelial SK_Ca_ and IK_Ca_ and VSMC BK_Ca_ activation resulting in the hyperpolarization and relaxation of VSMCs [4, 5]. DHA and DHA-derived EDPs activate BK_Ca_ channels present in VSMCs from porcine coronary [25] and rat coronary arteries [62, 63]; with EDPs reported to be 1000 times more potent in activating BK_Ca_ compared to AA-derived EETs. Similarly, CYP450 metabolites of EPA, 17R, 18S-EpETEs, activate BK_Ca_ channels in rat cerebral and mesenteric arteries [26] as well as human pulmonary artery [60]. Consistent with these studies, our results demonstrate that DHA-mediated relaxations in both mesenteric artery and aorta have a component sensitive to the blockade of BK_Ca_ (Figs 4A and 5A). Our data is consistent with the ability of DHA to directly activate these channels as the relaxations are independent of the metabolic action of CYP450 (Fig 3A and 3C). In the mesenteric artery (but not the aorta), BK_Ca_ blockade also led to inhibition of EPA-induced relaxation (Fig 4B). It is conceivable that EpETEs derived by metabolism of EPA by CYP450 could contribute to this effect as inhibition of CYP450 also reduced EPA-mediated relaxation (Fig 3B). The lack of any effect on EPA-induced relaxation following BK_Ca_ inhibition in the aorta indicates that direct or indirect modulation of this channel by EpETEs does not occur in this artery; again demonstrating the heterogeneity in the vasodilator mechanisms of n-3 PUFA mediated responses, depending upon both the type of artery and the n-3 PUFAs used to evoke relaxation.

To date there are no reports of n-3 PUFAs activating the SK_Ca_ and IK_Ca_ channels involved in EDH mediated relaxations. Blockade of the SK_Ca_ channel did not modify the relaxation responses to DHA or EPA in either artery. It is worth noting that we preconstricted arteries with U46619 and that activation of TP receptors inhibits SK_Ca_ channel activity in rat cerebral [64] and mesenteric arteries [65]. Therefore, it is possible that any potential SK_Ca_ component of n-3 PUFA-mediated relaxation was masked. However, an entirely novel finding of this study is that IK_Ca_ blockade inhibits DHA-induced relaxation of rat mesenteric artery and aorta (Figs 4A and 5A respectively). Furthermore, IK_Ca_ also contributed to EPA-induced relaxation of rat mesenteric artery (Fig 4B). This was surprising as it has been previously reported that DHA inhibits IK_Ca_ currents [66] in human embryonic kidney (HEK) cells. We cannot fully explain this discrepancy, but arterial IK_Ca_ channels are restricted to signalling microdomains in the endothelium where activation of associated proteins regulates IK_Ca_-mediated hyperpolarization [67, 68]. It is possible that HEK cells lack these microdomains, and thus what we observe may reflect an indirect activation of IK_Ca_ by DHA observed only in native tissue.

The endothelium-independent vasodilation mechanisms of n-3 PUFAs in arteries have not been extensively studied and remain unclear. BK_Ca_ are predominantly expressed in VSMCs and as discussed earlier, DHA and n-3 PUFA metabolites have been found to activate BK_Ca_ [25, 62, 63]. These metabolites are generally reported to be produced by endothelium derived enzymes such as CYP450 epoxygenase, but DHA also directly activates BK_Ca_ channels in the VSMCs [62]. Our data supports the direct action on VSMCs through BK_Ca_, as there was a minimal role of endothelium-dependent mechanisms in n-3 PUFA-induced relaxations. However, other endothelium-independent mechanisms for n-3 PUFA induced relaxation have been reported, for example, via inhibition of calcium influx in sheep pulmonary artery [69]. Furthermore, n-3 PUFAs are known to activate protein kinases such as protein kinase G, as demonstrated in cardiac fibroblasts [70]. If n-3 PUFAs are involved in activation of protein kinase G in arteries, they would also indirectly activate BK_Ca_ [71–74] which would be consistent with our findings. n-3 PUFAs also activate protein kinase A in rat cardiac cells, epithelial cells and human adipocytes [75, 76]. Protein kinase A can also evoke vasodilation, through direct activation of vascular K_ATP_ [77], therefore it can be speculated that n-3 PUFAs could also have an indirect interaction with potassium channels through the modulation of protein kinases, presenting an avenue for future investigation.

## Conclusion

**Fig 6:**
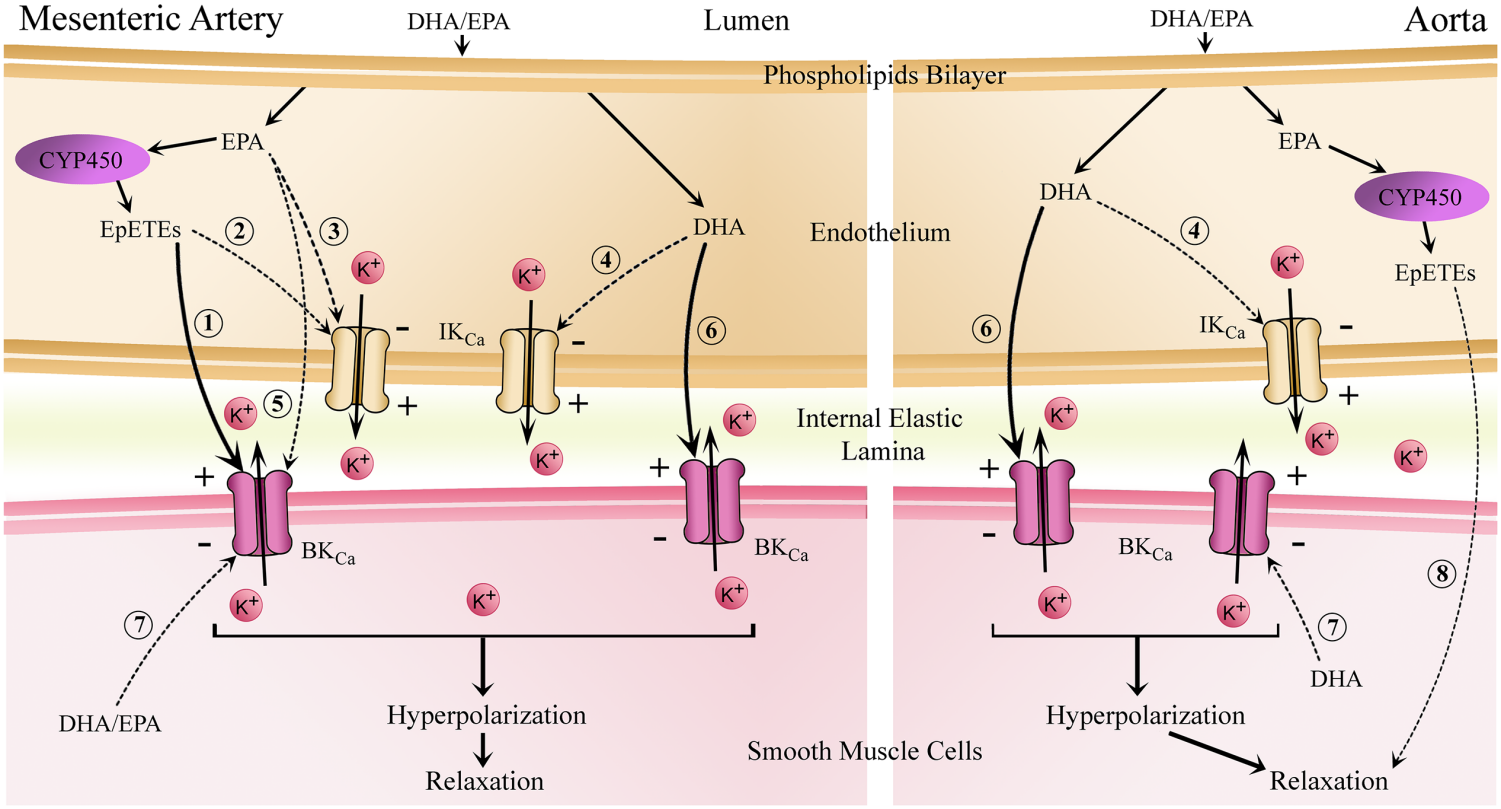
A schematic showing potential mechanisms involved in n-3 PUFA-induced relaxation of rat mesenteric artery and aorta preconstricted with U46619. Solid arrows represent pathways that have been previously investigated whereas dotted arrows represent hypothetical pathways based on our findings. In mesenteric arteries n-3 PUFAs enters the cytosol of the endothelium via diffusion from the plasma or release from the phospholipid bilayer via the activity of phospholipase A2 (PLA2); (1) EPA can be converted into EpETEs by CYP450, activating BK_Ca_. IK_Ca_ may be potentially activated by (2) EpETEs, (3) EPA and (4) DHA. (5) Similar to EpETEs, EPA might also be involved in the direct activation of BK_Ca_. (6) DHA directly activates BK_Ca_ (62). (7) Both n-3 PUFAs may enter the cytosol of VSMCs via diffusion or release from the phospholipid bilayer due to the activity of phospholipase A2 (PLA2) and directly activate BK_Ca_. (8) CYP450 derived metabolites of EPA such as EpETEs may induce vasodilation through K_Ca_ independent mechanisms which could involve other potassium channels such as K_ATP_.

The aim of this study was to characterise the mechanisms of DHA- and EPA-dependent vasodilation in rat conduit and resistance arteries. We demonstrate that endothelium has a minor role in these relaxations as confirmed by NO and COX not being involved in n-3 PUFA-induced relaxation and CYP450 metabolism only having a small effect. These findings are summarised in Fig 6 and they clearly demonstrate heterogeneity in the vasodilation mechanisms of n-3 PUFAs depending upon both the type of n-3 PUFA and the vascular bed. Similar to previous studies, BK_Ca_ was found to be involved in DHA- and EPA-induced relaxation [25, 26, 62, 63]. However, we also observed a novel role for IK_Ca_ in DHA- and EPA-induced relaxation of rat mesenteric arteries. Despite inhibition of a number of major vasodilator pathways, a large proportion of relaxation remained residual to these interventions indicating the presence of uncharacterised, endothelium-independent vasodilation mechanisms, which may involve other K^+^ channels and protein kinases [24, 50, 77, 78]. In conclusion, our study provides evidence of significant heterogeneity in the mechanisms of n-3 PUFA mediated relaxation in rat aorta and mesenteric artery along with a novel role for IK_Ca_. We believe these findings will be invaluable for the design of future vascular studies that involve the use of n-3 PUFAs.

## Supporting information

S1 File Supplementary data for: Characterisation of the vasodilation effects of DHA and EPA, n-3 PUFAs (fish oils), in rat aorta and mesenteric resistance arteries. Table A shows curve fit analysis for all experimental groups in each artery type Table B Shows curve fit analysis for pooled control data for DHA and EPA in each artery type.
(DOCX)
