# Supplementary material for: Characterisation of the vasodilation effects of DHA and EPA, n-3 PUFAs (fish oils), in rat aorta and mesenteric resistance arteries
Source: PLoS One. 2018 Feb 2;13(2):e0192484. doi: 10.1371/journal.pone.0192484 (PMC5796719; doi:10.1371/journal.pone.0192484)
Supplement: S1 File — Table A shows curve fit analysis for all experimental groups in each artery type Table B Shows curve fit analysis for pooled control data for DHA and EPA in each artery type. (DOCX) [file pone.0192484.s001.docx]

**Supplementary data for: Characterisation of the vasodilation effects of DHA and EPA, n-3 PUFAs (fish oils), in rat aorta and mesenteric resistance arteries**

**Roshan Limbu, Graeme S Cottrell and Alister J McNeish^*^**

Reading School of Pharmacy and ICMR, University of Reading, Reading, Berkshire, United Kingdom

**Results and discussion of supplemental data**

As well as data presented in the main manuscript we also analysed all experimental groups using generated curve fit data using (standard variable slope least squares fit based on the hill equation; Table A). However, as 30 µM was the highest concentration we could achieve without solubility or vehicle effects – this meant the maximal response was not always adequately defined – therefore, curve estimation prone to error therefore we analysed differences between groups using one way ANOVA and Bonferroni’s post-test as indicated in main article. However, we did pool control curve data from both aorta and mesenteric artery to see it there was an overall difference in either potency (EC_50_) or maximum response (E_max_ %) which demonstrated that there is no difference in the maximal response to omega-3 polyunsaturated fatty acid in either artery. DHA was significantly more potent that EPA in both arteries (Table B)

**Table A** summarises logEC_50_ and maximal relaxation (E_max_ % ) values obtained for each experimental group using standard variable slope least squares fit based on the hill equation in GraphPad Prism 5. N.D. indicates not determined as GraphPad could not produce an optimal curve fit due to nature of the data points, resulting in ambiguity for the values obtained. L-N indicates L-NAME. TRAM indicates TRAM-34 and Pax indicates Paxilline

| **Experiment** | **Type of artery** | **n-3 PUFA** | **Experimental**  **Condition** | **LOG EC_50_** | | **E_max_ (%)** | |
| --- | --- | --- | --- | --- | --- | --- | --- |
|  |  |  |  | **LOG EC_50_** | **SEM (±)** | **E_max_ (%)** | **SEM (±)** |
| **Endothelium removed** | Mesenteric  artery | DHA  *n=5* | Control | -5.79 | 0.04 | 92.71 | 2.77 |
|  |  |  | Endothelium removed | -5.56 | 0.05 | 95.80 | 4.56 |
|  |  | EPA  *n=6* | Control | -5.43 | 0.02 | 100.60 | 2.11 |
|  |  |  | Endothelium removed | -5.37 | 0.03 | 100.30 | 2.31 |
|  | Aorta | DHA  *n=7* | Control | -5.37 | 0.06 | 89.52 | 5.28 |
|  |  |  | Endothelium removed | -5.30 | 0.07 | 91.33 | 7.03 |
|  |  | EPA  *n=6* | Control | -5.25 | 0.03 | 94.19 | 2.13 |
|  |  |  | Endothelium removed | -5.11 | 0.05 | 94.72 | 5.18 |
| **L-NAME,**  **Indomethacin** | Mesenteric  artery | DHA  *n=5* | Control | -5.80 | 0.03 | 95.89 | 1.93 |
|  |  |  | L-N | -5.79 | 0.04 | 98.59 | 2.79 |
|  |  |  | L-N+  Indomethacin | -5.80 | 0.06 | 98.36 | 4.11 |
|  |  | EPA  *n=5* | Control | -5.56 | 0.06 | 101.10 | 5.43 |
|  |  |  | L-N | -5.68 | 0.06 | 99.46 | 4.99 |
|  |  |  | L-N+  Indomethacin | -5.58 | 0.08 | 102.40 | 7.35 |
|  | Aorta | DHA  *n=5* | Control | -5.30 | 0.05 | 90.08 | 4.29 |
|  |  |  | L-N | -5.22 | 0.07 | 89.00 | 5.98 |
|  |  |  | L-N+  Indomethacin | -5.20 | 0.07 | 87.54 | 5.74 |
|  |  | EPA  *n=5* | Control | -5.31 | 0.05 | 93.34 | 3.03 |
|  |  |  | L-N | -5.27 | 0.04 | 92.13 | 2.12 |
|  |  |  | L-N+  Indomethacin | -5.25 | 0.03 | 92.41 | 1.32 |
| **Clotrimazole** | Mesenteric  artery | DHA  *n=5* | Control | -6.34 | 0.06 | 98.90 | 2.81 |
|  |  |  | Clotrimazole | -6.16 | 0.06 | 96.84 | 2.76 |
|  |  | EPA  *n=5* | Control | -6.20 | 0.03 | 94.80 | 1.94 |
|  |  |  | Clotrimazole | -5.56 | 0.06 | 106.70 | 5.41 |
|  | Aorta | DHA  *n =5* | Control | -5.41 | 0.08 | 86.80 | 4.73 |
|  |  |  | Clotrimazole | **N.D.** | **N.D.** | 77.76 | 2.07 |
|  |  | EPA  *n=5* | Control | -5.00 | 0.06 | 97.05 | 9.89 |
|  |  |  | Clotrimazole | -4.93 | 0.44 | 77.89 | 24.04 |
| **L-NAME,**  **Apamin, TRAM-34, Paxilline** | Mesenteric  artery | DHA  *n=5* | Control | -5.87 | 0.04 | 96.66 | 2.72 |
|  |  |  | L-N | -5.88 | 0.04 | 97.65 | 3.36 |
|  |  |  | L-N+  Apamin | -5.82 | 0.04 | 96.34 | 3.10 |
|  |  |  | L-N+Apamin  +TRAM | -5.68 | 0.04 | 96.19 | 3.04 |
|  |  |  | L-N+Apamin  +TRAM+Pax | -5.37 | 0.06 | 91.20 | 5.31 |
|  |  | EPA  *n=5* | Control | -5.41 | 0.02 | 99.13 | 1.72 |
|  |  |  | L-N | -5.44 | 0.04 | 100.2 | 4.77 |
|  |  |  | L-N+  Apamin | -5.43 | 0.03 | 98.31 | 3.38 |
|  |  |  | L-N+Apamin  +TRAM | -5.34 | 0.06 | 99.94 | 6.06 |
|  |  |  | L-N+Apamin  +TRAM+Pax | -5.11 | 0.07 | 96.29 | 8.59 |
|  | Aorta | DHA  *n=5* | Control | -5.25 | 0.04 | 87.82 | 3.60 |
|  |  |  | L-N | **N.D.** | **N.D.** | 77.73 | 2.10 |
|  |  |  | L-N+  Apamin | **N.D.** | **N.D.** | 78.76 | 2.72 |
|  |  |  | L-N+Apamin  +TRAM | -5.28 | 0.06 | 71.98 | 4.28 |
|  |  |  | L-N+Apamin  +TRAM+Pax | -5.07 | 0.08 | 62.20 | 6.75 |
|  |  | EPA  *n=5* | Control | -5.19 | 0.06 | 91.54 | 5.08 |
|  |  |  | L-N | -5.25 | 0.03 | 90.74 | 1.29 |
|  |  |  | L-N+  Apamin | -5.24 | 0.03 | 89.89 | 1.56 |
|  |  |  | L-N+Apamin  +TRAM | -5.19 | 0.03 | 93.41 | 3.12 |
|  |  |  | L-N+Apamin  +TRAM+Pax | -5.10 | 0.05 | 91.16 | 4.72 |

**Table B** summarises logEC_50_ and maximal relaxation (E_max_ % ) values obtained for pooled control data for DHA and EPA in aorta and mesenteric arteries using standard variable slope least squares fit based on the hill equation in GraphPad Prism 5. Data were analysed using two-tailed t-test *P<0.05 indicates a significant difference from DHA in the artery studied. N.B where curve fitting was ambiguous or interrupted data was excluded

| **Type of artery** | **n-3 PUFA** | **LOG EC_50_** | | **E_max_ (%)** | |
| --- | --- | --- | --- | --- | --- |
|  |  | **EC_50_** | **SEM (±)** | **E_max_ (%)** | **SEM (±)** |
| Mesenteric  artery | DHA  *n=19* | \| -5.95 \|  \| \| --- \| --- \| | 0.05 | 96.51 | 1.01 |
|  | EPA  *n=20* | \| -5.66* \|  \| \| --- \| --- \| | 0.08 | 98.33 | 0.82 |
| Aorta | DHA  *n=18* | \| -5.33 \|  \| \| --- \| --- \| | 0.05 | 91.22 | 1.52 |
|  | EPA  *n=17* | \| -5.17* \|  \| \| --- \| --- \| | 0.06 | 94.39 | 1.48 |
